# From natural assemblages to synthetic communities in the *Lupinus* microbiome

**DOI:** 10.3389/fpls.2026.1891479

**Published:** 2026-07-31

**Authors:** Maite Ortúzar, Víctor Formariz, Jhon Alexander Suescún-Sepúlveda, Marta González-Hernández, Raúl Riesco, Ruben Garrido-Oter, Martha E. Trujillo

**Affiliations:** 1 Departamento de Microbiología y Genética, University of Salamanca, Salamanca, Spain; 2 Department of Plant-Microbe Interactions, Max Planck Institute for Plant Breeding Research, Cologne, Germany; 3 Cluster of Excellence on Plant Sciences, Düsseldorf, Germany; 4 Earlham Institute, Norwich, United Kingdom

**Keywords:** *lupinus* microbiome, metagenomics, plant-microbe interactions, root microbiota, synthetic communities, transcriptomics

## Abstract

**Introduction:**

Plant roots harbour complex microbial communities that enhance nutrient acquisition, stress tolerance, and pathogen defence, yet their assembly and functional dynamics remain incompletely understood.

**Results:**

In this work, we isolated over 700 bacterial strains from wild *Lupinus angustifolius* across multiple compartments and soil types, capturing both dominant and rare bacterial taxa. Using co-occurrence network analysis, we selected representative strains to assemble synthetic communities (SynComs) of varying complexity, which were inoculated under sterile and non-sterile conditions. Plants were inoculated with SynComs of increasing complexity under both non-sterile soil and gnotobiotic conditions. SynCom inoculation reshaped root-associated microbiota, moderately influenced the rhizosphere, and had limited impact on bulk soil communities. Increasing SynCom complexity enhanced plant growth and triggered host transcriptional responses involving hormone signaling, defence pathways, and metabolic reprogramming.

**Discussion:**

These findings indicate that soil-driven filtering and microbial interactions govern microbiome assembly and plant responses. Incorporating taxa with distinct ecological roles, including low-abundance members, improves SynCom functionality and advances understanding of plant-microbe interactions in natural and agricultural systems.

## Introduction

In natural environments, microorganisms are found in multispecies communities. These communities can range from only a few to, as seen in soil- and plant-associated microbiomes, hundreds of different species (Bulgarelli et al., 2013; Flemming and Wuertz, 2019). Plant roots interact with numerous microorganisms drawn from the surrounding soil microbiome, forming organized communities referred to as the root microbiota (Sokol et al., 2022). These communities provide the host plant with beneficial functions, including protection against pathogens and nutrient mobilization (Berg et al., 2014; Fitzpatrick et al., 2019; Feng et al., 2024; Li et al., 2026). Comparative analyses across terrestrial plants reveal clear differentiation in microbial profiles based on host species. Such patterns are thought to arise from microbiota assembly processes shaped by soil characteristics, climate, and plant-specific root exudates (Bai et al., 2015; Durán et al., 2018; Fitzpatrick et al., 2018; Wippel et al., 2021; Dai et al., 2025).

Despite these advances in understanding microbiome composition and assembly, experimental studies of plant-microbe interactions have historically relied on simplified systems. Until recently, most studies and downstream applications focused on single strains interacting with plants under sterile conditions (Karkaria et al., 2021). However, natural environments are not sterile, and introduced strains must compete with the resident microbiome, often leading to the failure of single-strain inoculants in the field (Baez-Rogelio et al., 2017). As an alternative, synthetic microbial communities (SynComs) are engineered to function as cohesive consortia, enhancing their survival and efficacy in non-sterile environments (Vorholt et al., 2017). Accordingly, SynComs provide a powerful framework to investigate microbiome functions and both plant-microbe and microbe-microbe interactions (Carlström et al., 2019; Ma et al., 2021; Emmenegger et al., 2023).

The use of host-specific SynComs, where community members are deliberately selected based on the study objectives, enables detailed functional characterization of individual taxa (Northen et al., 2024). This strategy allows for the dissection of both plant-associated and intra-community interactions under controlled conditions (Finkel et al., 2020; Wippel et al., 2021; Zhang et al., 2025). Importantly, SynComs also facilitate the inclusion of low-abundance microorganisms from the native plant microbiome, which may play critical ecological roles despite their minimal relative abundance (Compant et al., 2019).

To inform the design and interpretation of such communities, culture-independent approaches have been instrumental in advancing terrestrial microbiome research. These methods enable the characterization of microbial taxonomic composition via amplicon sequencing and the inference of functional potential through metagenomic and transcriptomic analyses (Zhang et al., 2015; Whelan et al., 2020). However, a major limitation remains as most microorganisms are not represented in culture collections, hindering their study in isolation or within defined consortia. This constraint directly limits the development of representative SynComs, as uncultured taxa cannot be incorporated into experimentally tractable communities. Consequently, establishing comprehensive culture collections is essential for the direct investigation of microbial associations and functional processes (Lebeis et al., 2012; Bai et al., 2015).

**Figure 1:**
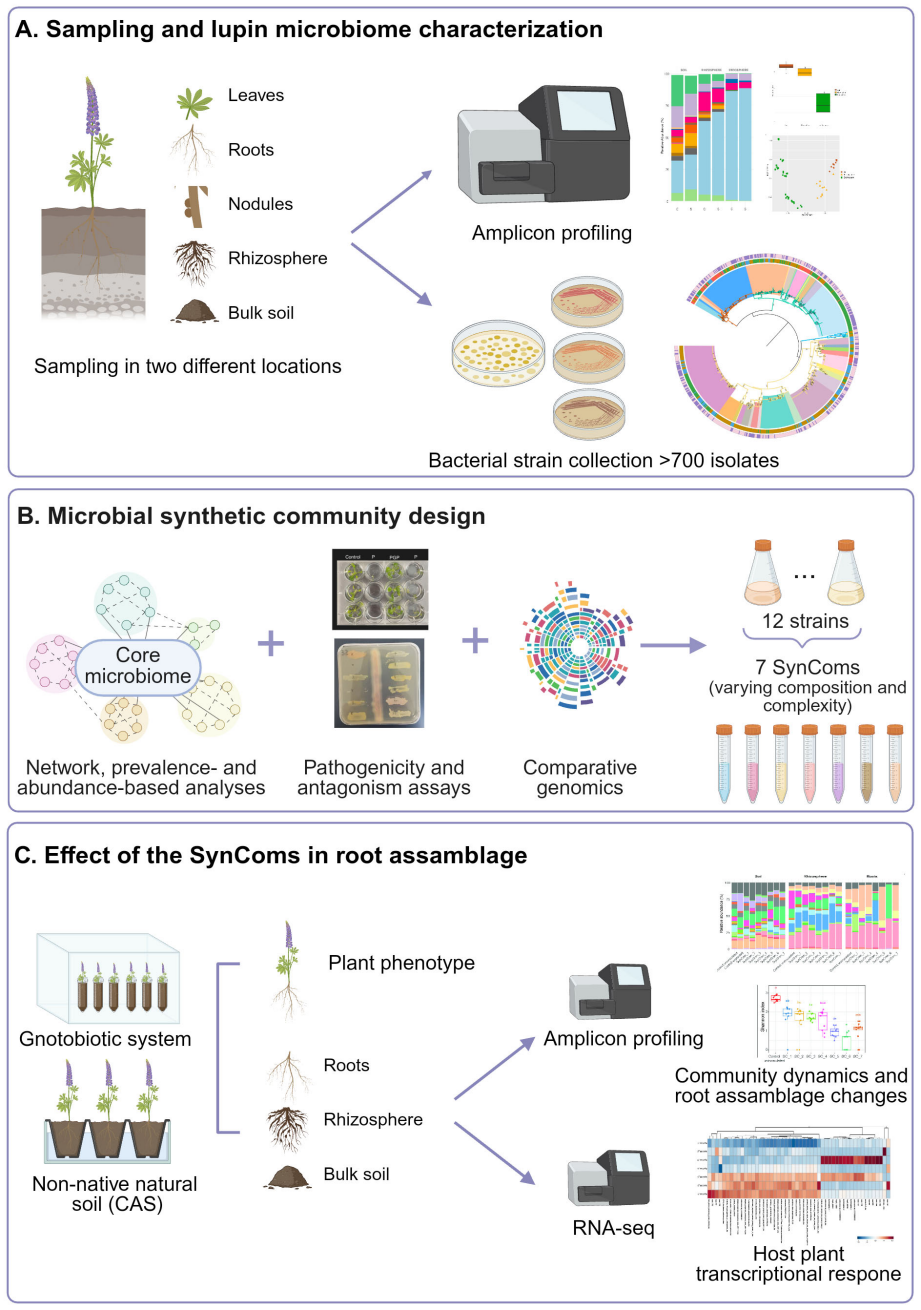
Overview of the experimental design of the study. **(A)** Plant sampling, isolation and identification of the microbiota associated to *L. angustifolius*. **(B)** Design and assemblage of bacterial synthetic communities (SynComs). **(C)** Inoculation of SynComs into sterile substrate and natural soil, followed by analysis of plant compartments using 16S rRNA gene amplicon sequencing and RNA-seq. Created with BioRender. https://biorender.com/5zocbp1.

Building on culture-independent data (Ortúzar et al., 2024), the aim of this work was to establish a diverse collection of bacterial isolates from the rhizosphere and tissues of wild *Lupinus angustifolius* capturing both dominant and low-abundance members of the microbiome. Using this collection, and guided by co-occurrence network analysis, we selected representative strains spanning distinct taxonomic groups to assemble synthetic communities for plant inoculation. The performance of these SynComs was evaluated in both gnotobiotic systems and a non-sterile agricultural soil distinct from the native lupin habitat, allowing us to assess microbial assembly, plant responses, and the ecological transferability of SynComs across contrasting microbial and edaphic environments (**Figure 1**).

We hypothesize that increasing SynCom complexity was predicted to promote plant growth and induce stronger effects on host gene expression and microbial assembly. In addition, taxa occupying central positions in the microbial co-occurrence network would have a greater impact on the community assembly than those with peripheral roles. To test these predictions, we compared SynCom performance across both gnotobiotic and non-sterile soil systems, providing a direct assessment of microbiome assembly and plant responses under contrasting microbial and edaphic conditions.

## Materials and methods

### Isolation and identification of bacteria from rhizosphere and plant tissues

*Lupinus angustifolius* plants and rhizosphere samples (30 per site) were collected in spring from two fields in Spain: Cabrerizos (40° 58’ 38.9” N; 5° 35’ 47.7”W) and Salamanca (40° 57’ 38.7”; N 5° 41’ 34.8” W). Plants were sampled at 8–9 weeks of age. While both sites are in the same region and experience similar climatic conditions, they differ markedly in soil physicochemical properties, providing contrasting edaphic environments.

Plant and rhizosphere samples were processed separately. Rhizosphere samples were processed as described by Thiergart et al. (2020), and serial dilutions (10–^4^ to 10^-10^) prepared. Roots and nodule processing followed the protocol described previously (Benito et al., 2022). Leaf-associated bacteria were isolated using three different treatments: (i) Leaves were surfaced sterilized by immersing in ethanol (70% v/v) for 1 min, transferred to 3.5% (w/v) sodium hypochlorite solution for 1 min and rinsed five times with sterile distilled water; (ii) leaves were shaken in PBS buffer (8 g/L NaCl, 0.20 g/L KCl, 1.44 g/L Na_2_HPO_4_, and 0.24 g/L KH_2_PO_4_) for 5h and the wash water was serially diluted and plated; and (iii) unsterilized leaves. Surface-sterilized and unsterilized leaves were homogenized with a sterile pestle.

Serial dilutions, wash suspensions, or tissue homogenates obtained from all sample types (rhizosphere, roots, nodules and leaves) were plated onto Reasoner’s R2A (R2A) (Reasoner and Geldreich, 1985), yeast mannitol (YMA) (Vincent, 1970), International Streptomyces Project 2 (ISP2) (Shirling and Gottlieb, 1966), peptone yeast extract (PYE) (Shirling and Gottlieb, 1966), starch casein (SCA) (Küster and Williams, 1964) and nutrient agar (NA) (Cross, 1968). All plates were incubated at 28 °C and checked every 2 days for colony emergence. Colonies were picked for up to 2 months after inoculation, and all isolates were subsequently stored in 20% (v/v) glycerol at -80 °C.

Bacterial genomic DNA was extracted with the REDExtract-N-Amp Plant PCR kit 1(Sigma) and the 16S rRNA gene was amplified and sequenced (SF1 5’- AGAGTTTGATCMTGGCTCAG-3’ and 1522R 5’- AAGGAGGTGWTCCARCC-3’) as previously described (Trujillo et al., 2010). Pairwise similarities between all the isolates were calculated using EzBioCloud (v2018.5) (Chalita et al., 2024) and aligned with Clustal X software (v2.0) (Clark et al., 2016). Evolutionary distances were calculated with Kimura’s two- parameter model and phylogenetic reconstruction at family level was done with the maximum- likelihood algorithm using the MEGA platform (v7) (Kumar et al., 2016).

### Pathogenicity assays

*Arabidopsis thaliana* Col-0 was used as a rapid and standardized model to screen the collection of more than 700 bacterial strains for potential phytopathogenicity before selecting strains for inclusion in the SynComs. Seeds were surface sterilized and germinated for five days at room temperature on MS medium (Murashige and Skoog, 1962) supplemented with sucrose 1% (w/v) and agar 1% (w/v) as described previously (Ortúzar et al., 2020). After 7 days, plantlets were transferred to 12-well plates, each containing 3 mL of the same medium and one plant per well. Test strains were cultured on ISP2 or R2A agar for five days at 28 °C. Bacterial suspensions (10^9^ UFC/mL) were prepared in saline solution (0.85% w/v) and used to inoculate 10 µL on each plant. All strains were tested in triplicate and control plants were inoculated with saline solution. Plants were incubated in a plant growth chamber with a 16/8h photoperiod program, with a constant temperature of 22 °C during the day and 19 °C at night, and 60% relative humidity. Plants were monitored during 14 days for any signs of tissue damage and compared to control plants.

### Selection criteria and design of synthetic microbial communities (SynComs)

We defined a root-associated lupin core microbiome using a prevalence- and abundance-based framework derived from 16S rRNA gene profiling (**Supplementary Figure 1**). Taxa were classified as core members if they were detected in at least 90% of the samples at each location and exhibited a relative abundance exceeding 0.1%. To further assess the ecological significance of these taxa, co-occurrence network analysis was conducted. This analysis identified three core-hub-associated (*Pseudomonas*, *Rhizobium* and *Variovorax*), five peripheral (*Bradyrhizobium*, *Caulobacter*, *Pseudoxanthomonas*, *Stenotrophomonas*, *Streptomyces*), and three satellite genera (*Kribbella*, *Micromonospora* and *Sphingopyxis*). Twelve representative strains from these genera were then randomly selected from the collection for inoculation experiments. The strains were combined into seven different synthetic communities ranging from one up to twelve strains (**Table 1**).

**Table 1:** Bacterial strains and their combinations in synthetic communities used for plant inoculation.

		Synthetic community composition						
Strain code	Identification	SC-1	SC-2	SC-3	SC-4	SC-5	SC-6	SC-7
CAR08	*Bradyrhizobium* sp.	✓		✓		✓		✓
Lupac 08	*Micromonospora* sp.		✓	✓			✓	✓
LARHSG274	*Caulobacter* sp.				✓	✓	✓	✓
LARHCF250	*Kribbella* sp.				✓	✓	✓	✓
LARHCG127	*Pseudomonas* sp.				✓	✓	✓	✓
LARHCG66	*Pseudoxanthomonas* sp.				✓	✓	✓	✓
LARHSG275	*Rhizobium* sp.				✓	✓	✓	✓
LARHCG72	*Sphingopyxis* sp.				✓	✓	✓	✓
LARHCG68	*Stenotrophomonas* sp.				✓	✓	✓	✓
LARHCF249	*Streptomyces* sp.				✓	✓	✓	✓
LARHCF252	*Streptomyces* sp.				✓	✓	✓	✓
LARHSF232	*Variovorax* sp.				✓	✓	✓	✓
Total number of strains in SynComs		1	1	2	10	11	11	12

Before plant inoculation, all strains were tested for mutual growth inhibition. Antagonism assays were performed by co-culturing all 12 strains on TSA or TY agar. Plates were incubated at 28 °C for up to 15 days to allow sufficient time for slow-growing strains to develop. The plates were then visually inspected for signs of growth inhibition.

### DNA extraction, genome sequencing and analysis of the selected strains

DNA extraction for genome sequencing was performed using the Wizard Genomic DNA Purification kit (Promega) using manufacturer’s instructions. Genomic DNA was randomly fragmented, end-repaired, A-tailed, and ligated with Illumina adapters to prepare libraries using the TruSeq kit, as per the manufacturer’s instructions. Sequencing was carried out on the Illumina MiSeq (300 pb paired-end) platform (Novogene UK), achieving a minimum depth of 100X coverage. The raw sequencing data was processed, after filtering, genome assembly was carried out using SOAPdenovo (Li et al., 2010), SPAdes (Bankevich et al., 2012), and ABySS (Simpson et al., 2009), and the best assembly, containing the fewest scaffolds, was selected using CISA software (Lin and Liao, 2013).

Coding sequences (CDSs) were predicted using GeneMarkS (Besemer et al., 2001), repetitive sequences detected using RepeatMasker, and tandem repeats analysed with Tandem Repeat Finder (TRF) (Benson, 1999). tRNAs were predicted using tRNAscan-SE (Schattner et al., 2005), rRNAs were identified using rRNAmmer, and snRNAs were identified using BLAST against the Rfam database 12.0 (Nawrocki et al., 2015). Functional annotation was performed using KEGG (Kanehisa and Goto, 2000) and PLaBAse (Patz et al., 2021) databases.

### SynCom inoculation of *Lupinus angustifolius* under gnotobiotic conditions

To assess the root colonization potential of the SynComs, a gnotobiotic plant inoculation experiment was conducted using the FlowPot system, ensuring even distribution of bacterial strains on roots and cultivation under sterile conditions (Kremer et al., 2018; Wippel et al., 2021). Each strain was grown individually in liquid medium TSB (BD) or TY broth (DSMZ medium 1143), as appropriate, and incubated at 28 °C and 200 rpm. Bacterial strains were mixed in equal amounts to reach an OD_600_ of 1. The suspension mixtures were added to Broughton and Dilworth (BD) medium (Broughton and Dilworth, 1971) without nitrogen to a final OD_600_ of 0.02 and each FlowPot was then flushed with 50 mL of the inoculum (BD medium/SynCom mix). Single strain and SynCom aliquots (50 µL and 200 µL) were stored at -80 °C for reference DNA sequencing (input).

Lupin seeds collected from wild plants were surface sterilized for 20 min in 3.5% sodium hypochlorite and rinsed five times with sterile distilled water. The seeds were germinated on water-agar plates (1% w/v) at room temperature in the dark and transferred after 5 days to the flushed FlowPots containing a matrix of sterile peat and vermiculite (2:1). Pots were incubated for six weeks at 21 °C/16 h light and 19.5°/8 h dark and 60% relative humidity. A detailed scheme of the plant treatments and corresponding controls is given in **Supplementary Figure 2A**.

Plants were harvested after eight weeks as described previously (Thiergart et al., 2020). Rhizosphere and root samples from each treatment were collected (9 biological replicates per sample type and treatment) for DNA extraction. The V5-V7 region of the 16S rRNA gene was amplified and sequenced for bacterial community profiling. For control pots without plants, substrate samples were also collected and processed using the same protocol to assess bacterial contamination. Plant growth parameters, including shoot length (cm), shoot fresh weight (g), root fresh weight (g), and nodule number were recorded at harvest. Root samples from all plants were also collected, immediately frozen in liquid nitrogen, and stored at -80 °C for subsequent RNA-seq analysis.

### SynCom inoculation of *Lupinus angustifolius* in soil

This experiment evaluated the capacity of the SynComs to colonize the host plant and their effect on the bacterial community in a soil (see below) different from the collection sites. Lupin seeds were surface sterilized and germinated as described above. A total of 120 seeds (fifteen seeds per treatment) were transferred to pots (3 L) containing CAS soil (Agricultural Soil from Cologne, Germany).

Bacterial strains were grown as described above. Biomass was centrifuged, washed, and resuspended in 10mM MgSO_4_ and adjusted to an OD_600_ of 0.02 (10^7^ cells/mL). Suspensions were mixed in equal ratios, and plantlets were inoculated with 1 mL of the bacterial cultures. In parallel, aliquots of each strain and of the different SynComs were stored at -80 °C for sequencing and used as reference samples (input) at the start of the experiment. Controls (soil pots and non-inoculated plants) were treated with the same amount of 10mM MgSO_4_ (5 mL per pot, 1 mL per plantlet). Plants were grown under greenhouse conditions for eight weeks under long-day cycles of (16h light/8h dark) and watered as needed. Soil, rhizosphere and root samples were collected as described previously (Thiergart et al., 2020). DNA was extracted and 16S rRNA gene profiling of the V5-V7 was amplified and sequenced. In addition, plant growth parameters such as root length and wet weight, shoot length and number of nodules, were recorded as previously done.

### DNA sequencing and 16S rRNA gene profiling

Total DNA from all soil samples was extracted using the FastDNA SPIN Kit for Soil (MP Biomedicals) according to the manufacturer’s instructions and the modified protocol described by Getzke et al. (2023). DNA from the SynCom inputs was isolated as described previously (Bai et al., 2015). Concentrations were measured and adjusted to 20 ng/µL for PCR reactions. V5-V7 bacterial variable region was targeted and amplified (primers 799F-1192R) for library preparation as described before (Durán et al., 2018). DNA sequencing was performed with Illumina MiSeq (Max Planck Genome Centre in Cologne, Germany).

Amplicon sequencing data from both experiments (soil and gnotobiotic conditions) were demultiplexed and analysed according to their barcode sequence using QIIME pipeline (Caporaso et al., 2010). Bacterial high-quality sequences were processed and ASV were taxonomically classified against the EzBioCloud database v2018.5 (Yoon et al., 2017).

### Plant transcriptome analysis

RNA extraction from the FlowPot plant roots (**Figure 1** and **S2A**) was done with RNeasy Plant Mini kit (Qiagen) following the manufacturer’s instructions. mRNA was purified from total RNA using poly-T oligo-attached magnetic beads. After fragmentation, the first strand cDNA was synthesized using random hexamer primers, followed by the second strand cDNA synthesis (Novogene UK). Sequencing was carried out with Illumina HiSeq3000 (Novogene UK). Raw sequencing reads were processed for quality control using Novogene in-house Perl scripts to remove low-quality reads, read containing adapters, and reads with poly-N. Reads were mapped to the *L. angustifolius* genome (GCA_001865875) using HISAT2 (v2.0.5) (Kim et al., 2019), which generates splice junction databases to improve mapping accuracy. Gene expression levels were quantified using featureCounts (v1.5.0-p3) (Liao et al., 2014). Differential expression analysis was performed using the DESeq2 package (Love et al., 2014), with statistical routines based on a negative binomial distribution. P-values were adjusted for multiple comparisons using the Benjamini-Hochberg method, with an adjusted P-value ≤ 0.05 considered significant. KEGG pathway enrichment analysis was performed using the clusterProfiler R package (Xu et al., 2024). KEGG pathways with a corrected P-value <0.05 were considered significantly enriched. Gene Set Enrichment Analysis (GSEA) was performed to assess whether predefined gene sets showed significant and consistent differences between biological conditions. The genes were ranked by their differential expression, and KEGG datasets were used to identify enriched gene sets, with the analysis conducted using the GSEA tool (Subramanian et al., 2005).

### Statistics and reproducibility

Plant experiments were independently repeated twice to ensure reproducibility. Individual plants served as biological replicates. A total of nine biological replicates per treatment were used for amplicon sequencing, and three biological replicates per treatment were used for RNA-seq analysis. Statistical analyses and visualization were performed with the R software (v4.5.3) (R Core Team, 2024), unless otherwise indicated. Amplicon diversity analysis was performed using QIIME v1.9.1 (de Mendiburu, 2023). *Beta* diversity was performed using Bray Curtis distance, a Permutational Multivariate analysis of Variance (PERMANOVA) test with 999 random permutations and Principal Coordinate Analysis (PCoA) (*vegan* v. 2.6-4) (Oksanen et al., 2022). All PERMANOVA results were significant with a P<0.001 unless otherwise specified. Bacterial community composition was represented as bar plots. Microbial abundances were compared with the nonparametric Mann–Whitney test or, in the case of multiple comparisons with the Kruskal–Wallis test, followed by a Tukey *post hoc* test for analysis. Microbial co-occurrence networks were inferred from the OTU abundance table using the SparCC algorithm as implemented in FastSpar (Watts et al., 2019). Pairwise correlations were calculated and their significance was assessed using bootstrap resampling (1000 permutations). Only robust and significant associations were retained (|r| ≥ 0.7 and p < 0.05) to construct the adjacency matrix. The resulting network was visualized and analysed in R using the package igraph v2.2.2 (Csardi and Nepusz, 2006; Csárdi et al., 2026), and exported for visualization in Gephi (Bastian et al., 2009). Topological roles of taxa within the network within-module connectivity (Zi) and among-module connectivity (Pi) were calculated. Modules were detected using a greedy modularity optimization algorithm implemented in igraph. Based on Zi and Pi thresholds (Zi = 2.5 and Pi = 0.62), nodes were classified as peripherals, connectors, module hubs, or network hubs (Guimerà and Amaral, 2005). All plots were represented using *ggplot2* v4.0.2 (Wickham, 2016).

## Results

### Bacterial strain collection associated to *Lupinus angustifolius*

In a previous study, we used metagenomic profiling to characterize the microbiome associated with *L. angustifolius* (Ortúzar et al., 2024). These analyses provided information on bacterial abundance and taxonomic composition across different plant compartments (rhizosphere, roots, nodules, and leaves). This information was used to design an isolation protocol aimed at maximizing the recovery of bacterial diversity, including both dominant and low-abundance taxa. This strategy combined multiple isolation media with extended incubation times, yielding a total of 722 strains that were isolated, purified, and stored in 20% (v/v) glycerol at -80 °C (**Supplementary Table 1**).

The highest number of strains (184) was isolated on R2A agar, followed by YMA (157), NA (109), PYE (94), SCA (42), and ISP2 (36) (**Supplementary Figure 3A**). By sampling location, 415 isolates were obtained from Cabrerizos and 307 from the Salamanca sites (**Supplementary Figure 3B**). The distribution of isolates across plant tissues and the rhizosphere also differed between locations (**Supplementary Figure 3C**).

### Taxonomic composition and distribution of the bacterial strain collection across plant compartments

**Figure 2:**
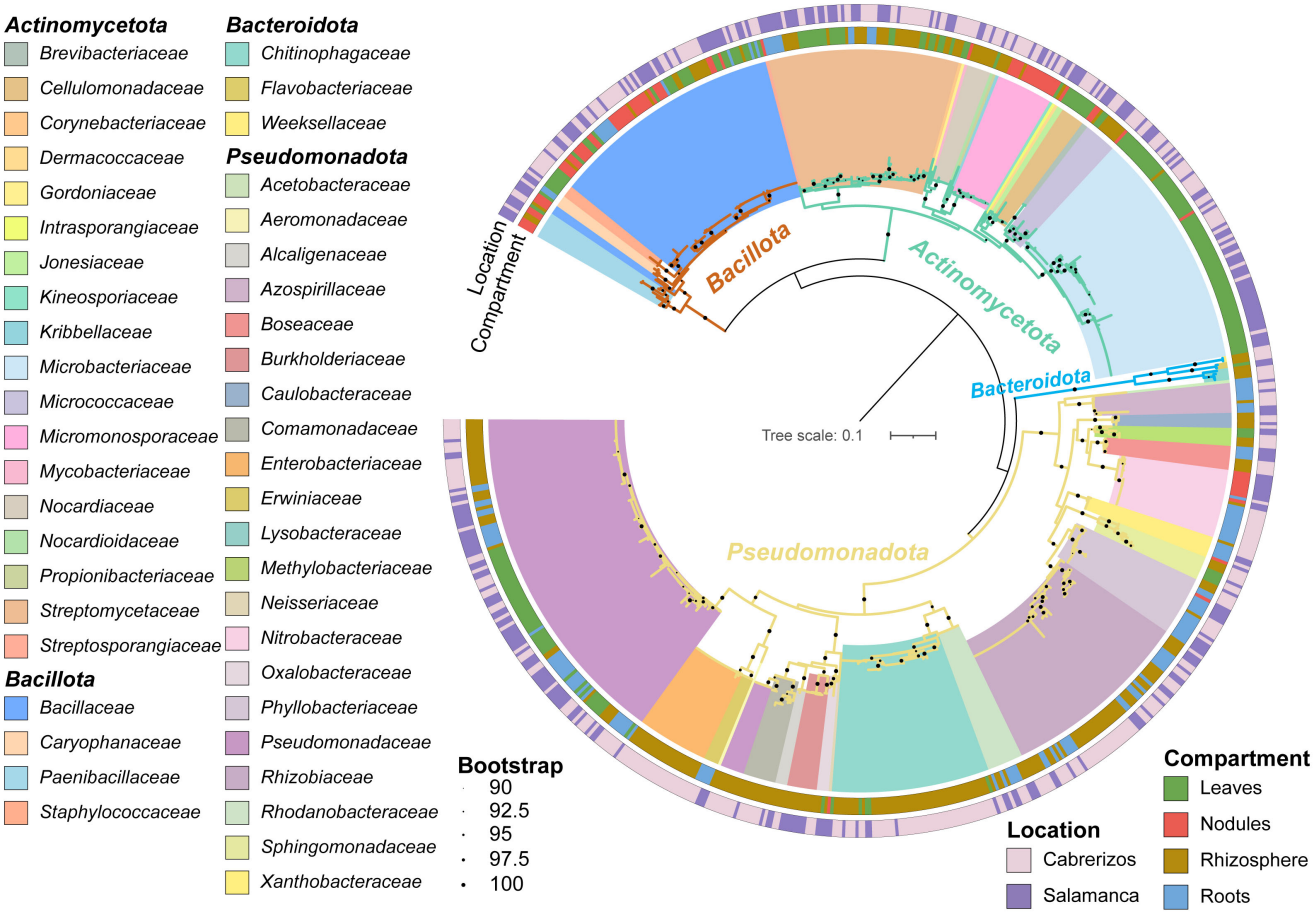
Maximum- likelihood phylogenetic tree based on 16S rRNA gene sequences. The tree shows the relationships at family level of all the strains isolated in this work in the different plant compartments (leaves, nodules, rhizosphere and roots). Also shows the distribution by location of the strains. Distances were calculated with the Kimura two- parameter model. The tree is based on 1034 nt. Bootstrap percentages ≥90 % (1,000 samplings) are shown at the nodes. Bar, 0.1 substitutions per nucleotide.

The strains were identified at the genus level using 16S rRNA gene sequencing, revealing 87 distinct genera and indicating a high level of microbial diversity. Their phylogenetic relationships at the family level, inferred from the 16S rRNA gene sequences of all isolates, are shown in **Figure 2**. Of these 87 genera, 52% were shared between the two sampling sites, while 48% were site-specific: 22% unique to Salamanca and 26% to Cabrerizos (**Supplementary Figure 4A**). A total of 30 taxa were shared among two or more plant compartments, including the rhizosphere. Only two genera, *Bacillus* and *Peribacillus*, were found in all samples (**Supplementary Figure 4B**). *Pseudomonas* was the most abundant genus (120 strains). Other prevalent genera included *Streptomyces* (60), *Agrobacterium* (36), *Bacillus* (31), *Pseudoclavibacter* (31), *Enterobacter* (24), *Microbacterium* (24), *Pseudoxanthomonas* (23) and *Stenotrophomonas* (22) (**Supplementary Figures 5–8**). The recovery frequency of each genus is represented in **Supplementary Figure 9** and **Supplementary Table 2**.

**Figure 3:**
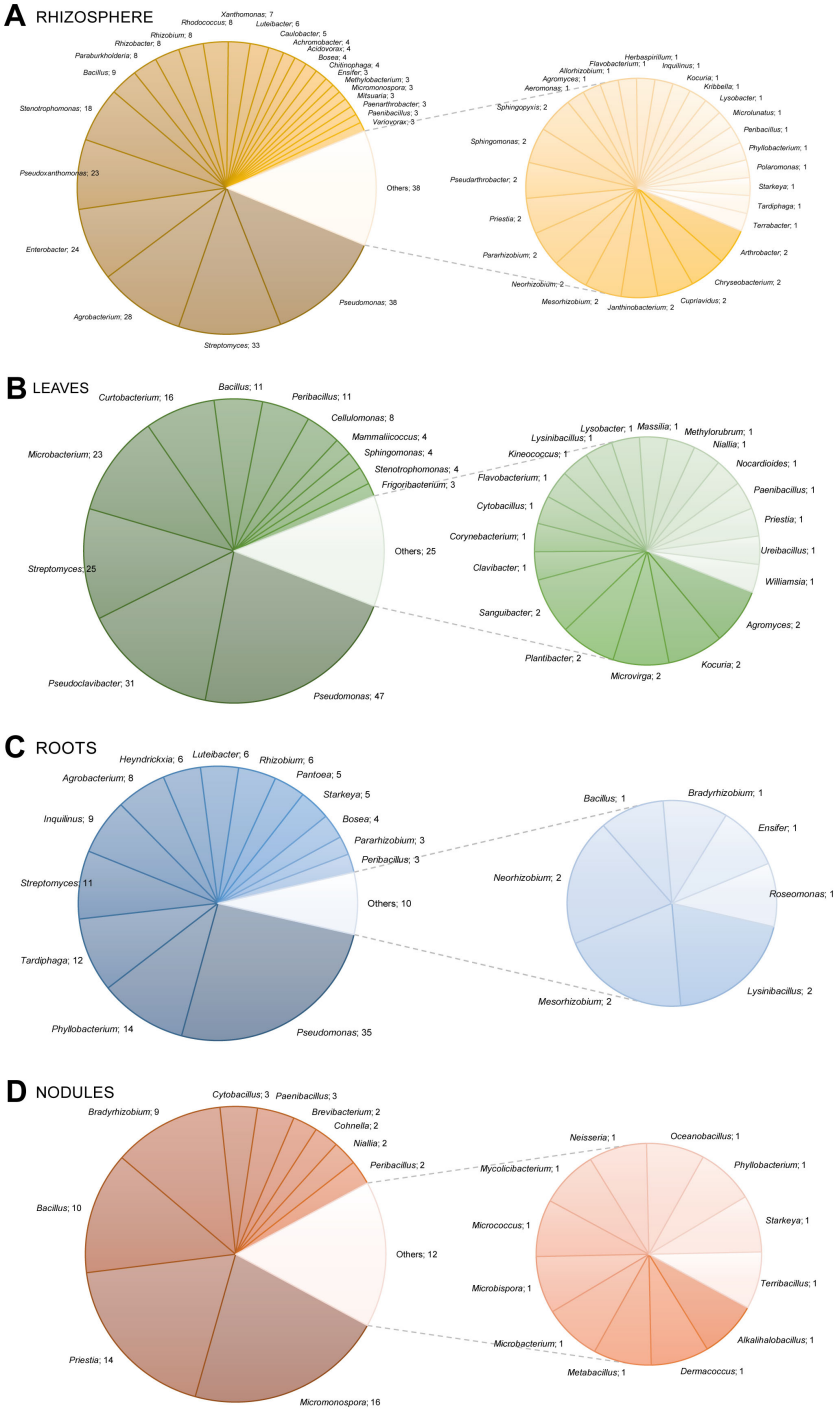
Bacterial genus diversity. Distribution of bacterial strains isolated in this study and identified at the genus level from the **(A)** rhizosphere, **(B)** leaves, **(C)** roots, and **(D)** nodules. The number of strains assigned to each genus is indicated next to the corresponding genus name.

As expected, the rhizosphere yielded the greatest number of strains, comprising 52 distinct genera, of which 38 were represented by a single isolate, underscoring the extensive diversity captured in the culture collection. The most frequently isolated genera were *Pseudomonas*, *Streptomyces*, *Agrobacterium*, *Enterobacter*, *Pseudoxanthomonas*, and *Stenotrophomonas*, whereas the remaining genera were represented by 1 to 9 strains (**Figure 3A**). Leaves were the second most abundant source, with 212 strains spanning 32 genera. Once again, *Pseudomonas* predominated, followed by *Pseudoclavibacter*, *Streptomyces*, *Microbacterium*, *Curtobacterium*, *Bacillus*, *Peribacillus*, and *Cellulomonas* (**Figure 3B**). Roots yielded 137 isolates representing 21 genera, with *Pseudomonas* as the most abundant, followed by *Phyllobacterium*, *Tardiphaga*, *Streptomyces*, *Inquilinus*, and *Agrobacterium* (**Figure 3C**). A total of 75 strains representing 22 genera were isolated from nodules, highlighting the remarkable diversity within this specialized tissue. The most abundant genera were *Micromonospora* and *Bradyrhizobium*, alongside *Priestia*, *Bacillus*, *Cytobacillus*, *Paenibacillus*, *Brevibacterium*, *Cohnella*, *Niallia*, and *Peribacillus* (**Figure 3D**). A detailed overview of genus distribution by location and the number of shared taxa between the rhizosphere and the different tissues is given in **Supplementary Figure 10**.

A complete list of isolates including their origin and taxonomic assignments, is provided in **Supplementary Table 1**. Microbial community composition inferred from 16S rRNA gene amplicon data (relative abundance >1%) was compared with cultured bacterial genera by integrating metagenomic and cultivation-based datasets. This analysis revealed that 28 genera detected by metagenomics above the 1% threshold were also successfully isolated (**Supplementary Figure 11**; **Supplementary Table 3**).

### Pathogenicity potential of the strain collection

*Arabidopsis thaliana* Col-O was used as a proxy for lupin to assess the potential pathogenicity of all isolates. After 14 days of growth in the presence of each strain, results were categorized as: “opportunistic pathogen” if plant tissue necrosis was observed, “non-pathogen” if plants developed without damage, and “plant growth promoter” (PGP) if plant growth was enhanced compared to the controls (detailed strain information in **Supplementary Table 1**). Overall, 55% of the strains were classified as non-pathogenic, 16% as PGP, and 29% as “opportunistic pathogens” (**Supplementary Figure 12A**).

The strains classified as “opportunistic pathogens” were isolated primarily from the rhizosphere (**Supplementary Figure 12B**) and belonged to the genera *Pseudomonas* (48 strains), *Enterobacter, Bacillus, Priestia, Pseudoclavibacter*, and *Pseudoxanthomonas* (**Supplementary Figure 12C**). These isolates were excluded from the synthetic bacterial communities used for plant inoculation.

Nevertheless, caution must be taken when considering these results. *Arabidopsis* should be viewed as a complementary model, not a substitute for testing on the host plant. Positive results on *Arabidopsis* can reveal conserved virulence traits, but negative results cannot reliably exclude phytopathogenicity toward lupin because of differences in host specificity, immune recognition, tissue biology, and the environmental cues that regulate bacterial virulence.

### Co-occurrence analysis and antagonistic assays

**Figure 4:**
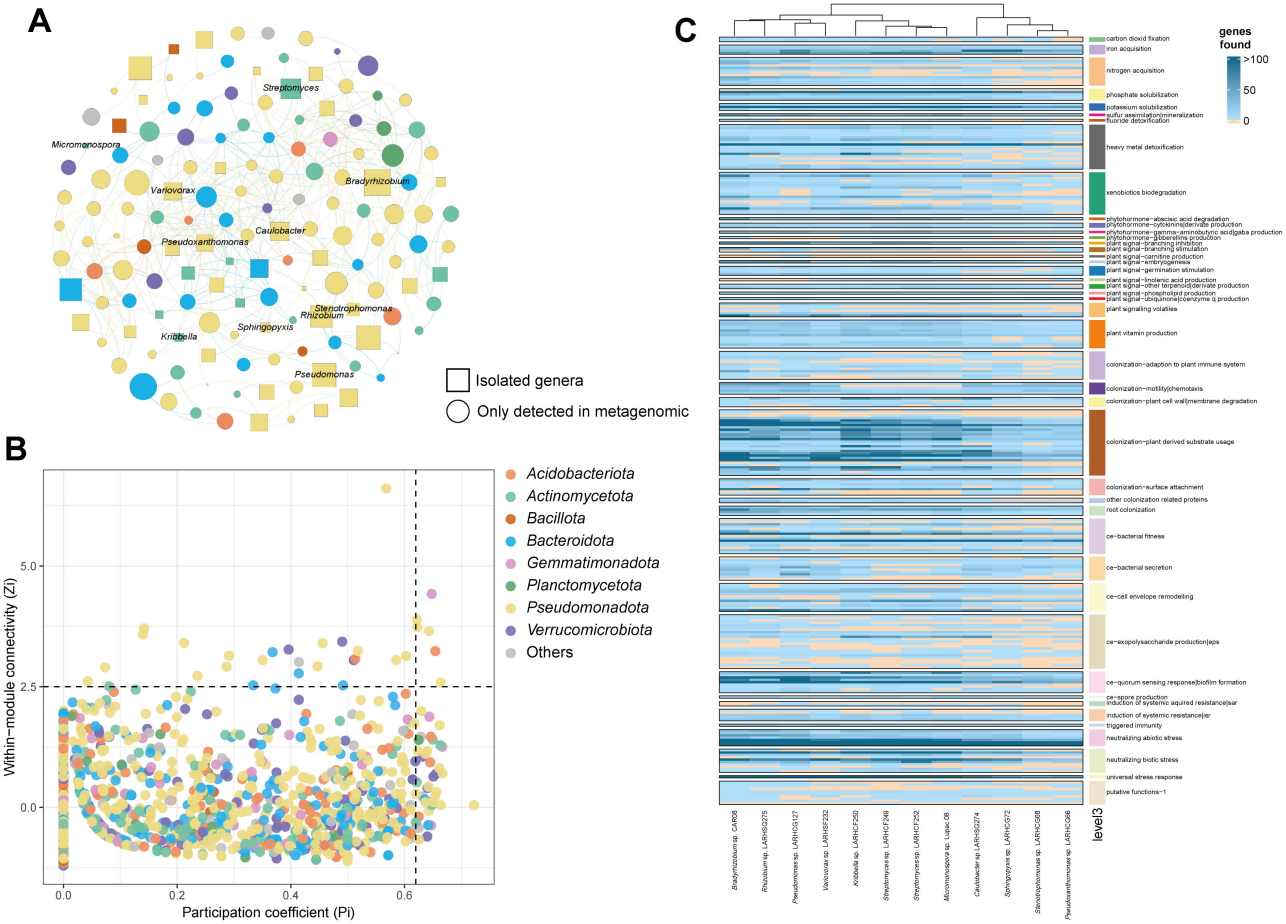
Co-occurrence network structure and functional potential of SynCom strains. **(A)** Co-occurrence network of the *L. angustifolius* microbiome based on amplicon sequencing data. Nodes represent ASVs and edges indicate significant correlations between taxa. Node size is proportional to relative abundance, and colours indicate taxonomic affiliation at the phylum level. ASVs corresponding to strains included in the SynCom are highlighted. **(B)** Topological role classification of ASVs based on within-module connectivity (Zi) and participation coefficient (Pi). Nodes are categorized as peripherals, connectors, module hubs, or network hubs according to their position in the network. **(C)** Heatmap showing the distribution of genes associated to plant growth promoting traits (PGPTs) across all bacterial strains included in the SynComs. Genome mining and PGPT categorization were performed using PLaBAse.

Co-occurrence network analysis of the *L. angustifolius* 16S rRNA gene amplicon data (Ortúzar et al., 2024) revealed a structured community with distinct topological roles, including highly connected taxa (hubs) and connector nodes linking different modules. Several taxa used to assemble the different SynComs occupied key positions with *Pseudomonas* and *Variovorax* identified as network and module hubs, respectively. In contrast, *Bradyrhizobium*, *Streptomyces*, and *Caulobacter* were classified as peripheral taxa (**Figures 4A, B**; **Supplementary Table 4**). Overall, the selected strains spanned a broad range of relative abundances, from dominant taxa such as *Bradyrhizobium* to low-abundance genera such as *Sphingopyxis* and *Kribbella*. Several SynComs also included a *Micromonospora* strain, extending its evaluation from previously described associations with rhizobia (Benito et al., 2017) to a broader host-associated microbial consortium.

No antagonistic interactions were observed between any of the strains under the conditions tested. All strain combinations exhibited normal growth without visible zones of inhibition on either TSA or TY agar (**Supplementary Figure 12D**).

### Genomic features and functional potential of the strains used for SynCom assemblage and inoculation

Genome sequences of the twelve bacterial strains used for SynCom assemblage and plant inoculation were obtained and used for species level identification and to screen for potential plant-growth promotion functions. Plant-associated gene categories were predicted using the PGPT-Pred module in PLaBAse, showing that all strains harboured numerous plant growth-promoting traits (PGPT, >200 hits per strain) (**Supplementary Table 5**). High gene abundances were observed for functions related to vitamin production, iron acquisition, plant colonization, phosphate solubilization, plant immune system adaptation, heavy metal detoxification and biotic stress neutralization (**Figure 4C**).

All strains contained multiple genes involved in the biosynthesis of vitamins B1, B3, B5, B6, B9 and B12, which are associated with root colonization (Riesco et al., 2022; Gonçalves et al., 2024). The highest copy numbers were detected in *Bradyrhizobium* sp. CAR08 (52) and *Streptomyces* LARHCF249 (42). Genes related to plant colonization, including those involved in carbohydrate transport and amino acid transport, surface attachment, motility and chemotaxis, were widely distributed and particularly abundant in Rhizobium sp. LARHSG275, *Bradyrhizobium* sp. CAR08, *Kribbella* sp. LARHXF250 and *Variovorax* sp. LARHSF232. In contrast, *Pseudoxanthomonas* sp. LARHCG66, *Stenotrophomonas* sp. LARHCG68, and *Sphingopyxis* sp. LARHCG72 showed lower PGPT abundance overall, except for genes related to heavy metal detoxification, iron acquisition, and biotic stress neutralization (**Figure 4C**; **Supplementary Table 5**).

### SynCom root colonization under sterile conditions

The capacity of root colonization and effects of the bacterial SynComs on lupin plants grown in a gnotobiotic system were assessed. Treated plants exhibited significantly enhanced growth compared to their respective controls. Among all combinations, SC-7 (12 strains), was the most effective in promoting plant growth. Statistical analyses, including Z-score standardization and ANOVA followed by Tukey’s *post-hoc* test (P<0.001), confirmed these differences. Inoculated plants showed improved growth relative to non-inoculated controls. These results were reproducible across both experimental replicates (**Supplementary Figure 13**; **Supplementary Table 6**).

To verify bacterial colonization, 16S rRNA gene profiling of rhizosphere and root samples was performed after harvest. All inoculated strains were detected, although their relative abundances varied across compartments (**Supplementary Figure 14A**; **Supplementary Table 7**). *Pseudomonas* sp. LARHCG127 was the most abundant strain, particularly in the roots of plants inoculated with SC-4, where it accounted for more than 90% of the community (**Supplementary Figure 14C**). However, its abundance decreased significantly when *Bradyrhizobium* sp. CAR08 and/or *M. lupini* Lupac08 were included in the consortia (SC-5, SC-6, and SC-7). *Sphingopyxis* sp. LARHCG72 was most abundant in the rhizosphere of SC-5, SC-6, and SC-7, while *Caulobacter* sp. LARHSG274 was the third most abundant strain, showing comparable levels in both roots and rhizosphere. In contrast, *Micromonospora* Lupac 08 and the two *Streptomyces* strains were nearly undetectable (**Supplementary Figures 14B, C**).

### Colonization and effect of the SynComs on the bacterial community of CAS soil

16S rRNA gene amplicon profiling of the soils where *L. angustifolius* naturally grows and of the CAS soil used in this experiment revealed marked differences in bacterial community composition. Despite physicochemical differences between the Cabrerizos and Salamanca soils (e.g. pH, nutrients, trace elements) (Ortúzar et al., 2024), their bacterial profiles were highly similar (**Supplementary Figure 15**; **Supplementary Table 8**). In contrast, CAS soil, which also differs markedly in its physicochemical composition from the native soils (Hou et al., 2021), exhibited a distinct microbial composition, characterized by a high relative abundance of *Arthrobacter*, *Gemmatimonadaceae*, *Lysobacter*, *Massilia*, *Polaromonas* and *Sphingomonas*. In the native soils, *Gaiella*, *Nitrospira*, *Pseudomonas*, *Streptomyces* and *Tepidisphaera* were among the predominant taxa.

**Figure 5:**
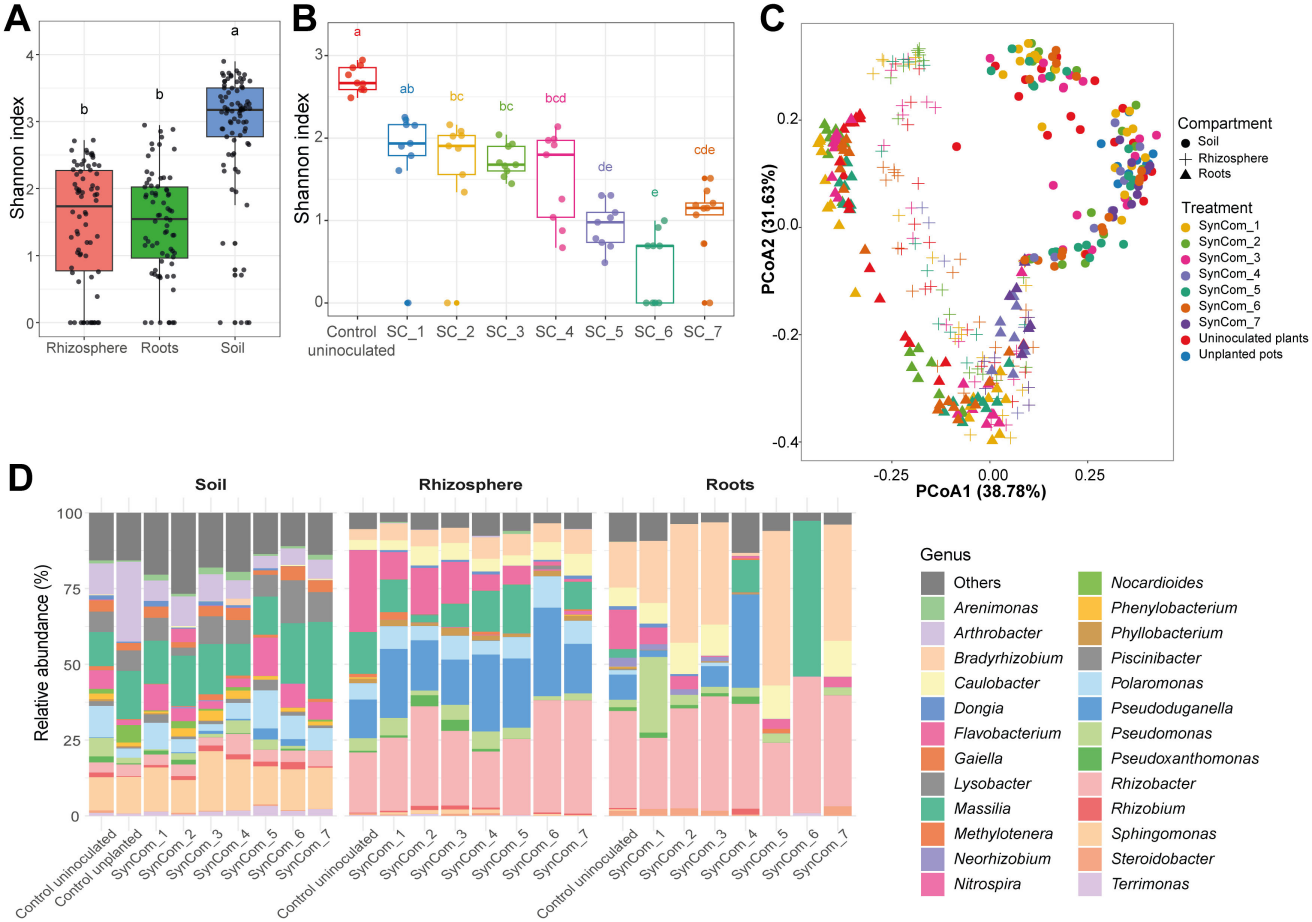
Effect of SynCom inoculation on the natural bacterial community associated with the host plant. **(A)** Box plots of Shannon index (alpha diversity) representation of bacterial communities in soil, rhizosphere and roots following inoculation with SynComs. **(B)** Alpha diversity comparison showing the impact of different SynComs on bacterial communities in the roots. Letters above the box plots indicate clustering of the sample at a significance of P<0.001. **(C)** Principal coordinate analysis (PCoA) of Bray-Curtis dissimilarities (beta diversity) showing variations in bacterial community composition across soil, rhizosphere, and roots inoculated with different SynComs. Group differences were evaluated using PERMANOVA, with P<0.001. **(D)** Bar plots representing the relative abundance of the most prevalent bacterial genera in the soil, rhizosphere and roots under the influence of the different SynComs.

Alpha diversity across all samples was assessed using the Shannon index. Combined analysis of both experiments revealed statistically significant differences among the three compartments. Soil samples exhibited the highest alpha diversity (P<0.0001), followed by the rhizosphere and roots (**Figure 5A**). Alpha diversity was also analysed separately for each plant compartment to evaluate the effect of SynCom inoculation. No significant differences were observed in soil or rhizosphere samples following inoculation with the different synthetic communities (**Supplementary Figures 16A, B**). In contrast, significant differences were detected in root samples (P<0.0001), with uninoculated control plants exhibiting the highest alpha diversity, followed by SC-1, SC-2, SC-3, SC-4, SC-6, SC-5, and SC-7 (**Figure 5B**).

Bacterial community composition across the three compartments (soil, rhizosphere, and roots) was compared using Principal Coordinates Analysis (PCoA) based on Bray–Curtis dissimilarity. Samples consistently clustered by compartment, with rhizosphere and root communities showing greater similarity to each other than to soil communities. No significant treatment effects were detected within compartments observed in soil, rhizosphere and root samples (PERMANOVA, Treatment|Compartment R^2^ = 0.078, p=0.001; **Figure 5C**).

The 16S rRNA gene profiling of the compartments inoculated with the different SynComs revealed that these microorganisms significantly influenced the assembly and composition of the root bacterial microbiota (**Figure 5D**; **Supplementary Table 9**). All introduced bacterial taxa were successfully detected in the sequenced inoculum (input) samples, which served as controls for both individual and combined strains.

In soil samples, bacterial composition remained stable across all treatments, with no significant differences observed following SynCom inoculation. In contrast, changes in bacterial composition were evident in the rhizosphere. The abundance of *Massilia* was strongly influenced by SynCom composition, with SC-2 and SC-6 reducing its relative abundance from over 9% in the control to less than 1%. Similarly, the abundance of *Flavobacterium* decreased from 14% to below 1% in treatments with SC-6 and SC-7. Across all SynCom-treated samples, the relative abundance of *Bradyrhizobium* was consistently higher than in the control, even in treatments where it was not introduced.

Root samples exhibited the greatest variability in response to SynCom inoculation in CAS soil. *Rhizobacter* was abundant across all samples and particularly enriched in plants inoculated with SC-3 and SC-6. The genus *Caulobacter* was nearly undetectable in treatments SC-4 and SC-6, coinciding with an increase in *Massilia* and the absence of *Bradyrhizobium* inoculation. *Pseudomonas* was detected in all samples and was highly enriched (>14%) in SC-2 treated plants. The most pronounced shifts were observed in the root samples treated with SC-6, where the bacterial community was completely displaced relative to the CAS soil control. In these samples, *Rhizobacter*, *Massilia*, and *Terrimonas* were highly enriched while *Bradyrhizobium* was undetectable. Finally, SynComs containing *Micromonospora* markedly reduced the abundance of *Pseudoduganella*, particularly when it was the only strain introduced (SC-2) (**Figure 5D**).

To distinguish the indirect effects of SynCom inoculation from the direct contribution of the introduced strains, we removed (*in silico*) the ASVs corresponding to the inoculated taxa and reassessed community composition. Under these conditions, both alpha and beta diversity remained stable, and the differences between rhizosphere and root communities became less pronounced (PERMANOVA Treatment|Compartment R^2^ = 0.063, p=0.001; **Supplementary Figure 17**). At the genus level in soil, more than 20% of the relative abundance in most samples corresponded to low abundance taxa (**Supplementary Figure 18**). In the rhizosphere, a consistent decrease in *Pseudomonas* abundance was observed across all treatments, along with a reduction in *Bradyrhizobium* in SC-5, SC-6, and SC-7 treatments, where it declined from approximately 8% to 0.5%. In roots, microbial profiles became highly consistent across treatments, showing limited variation. In the uninoculated control, more than 50% of the sequences were assigned to unclassified or low-abundance taxa, highlighting the susceptibility of roots to colonization by diverse environmental microorganisms. In contrast, inoculated plants exhibited stable colonization by SynCom members and their associated microbial networks. Notably, even after removing SynCom-derived ASVs, the overall root community structure remained highly similar to that observed when these taxa were included. This suggests that SynCom strains not only dominate root colonization but also shape the assembly of the surrounding microbial community and the occupation of the root niche (**Supplementary Figure 18**; **Supplementary Table 10**).

Inoculated plants exhibited improved growth compared to their respective controls (**Supplementary Figure 19**; **Supplementary Table 6**). Measurements of root and shoot length, fresh weight, and nodule number across both experiments identified SynCom SC-7 (12 strains) as the most effective in promoting plant growth. Significant differences among plant growth parameters were confirmed after Z-score standardization and analysis by ANOVA followed by Tukey’s *post hoc* test (P < 0.001). Notably, SynComs containing *Bradyrhizobium* sp. CAR08 and *Micromonospora* sp. Lupac 08 promoted significantly greater growth than those lacking these strains. These results were consistent across both experiments.

### Host transcriptomic shifts in response to SynCom complexity

**Figure 6:**
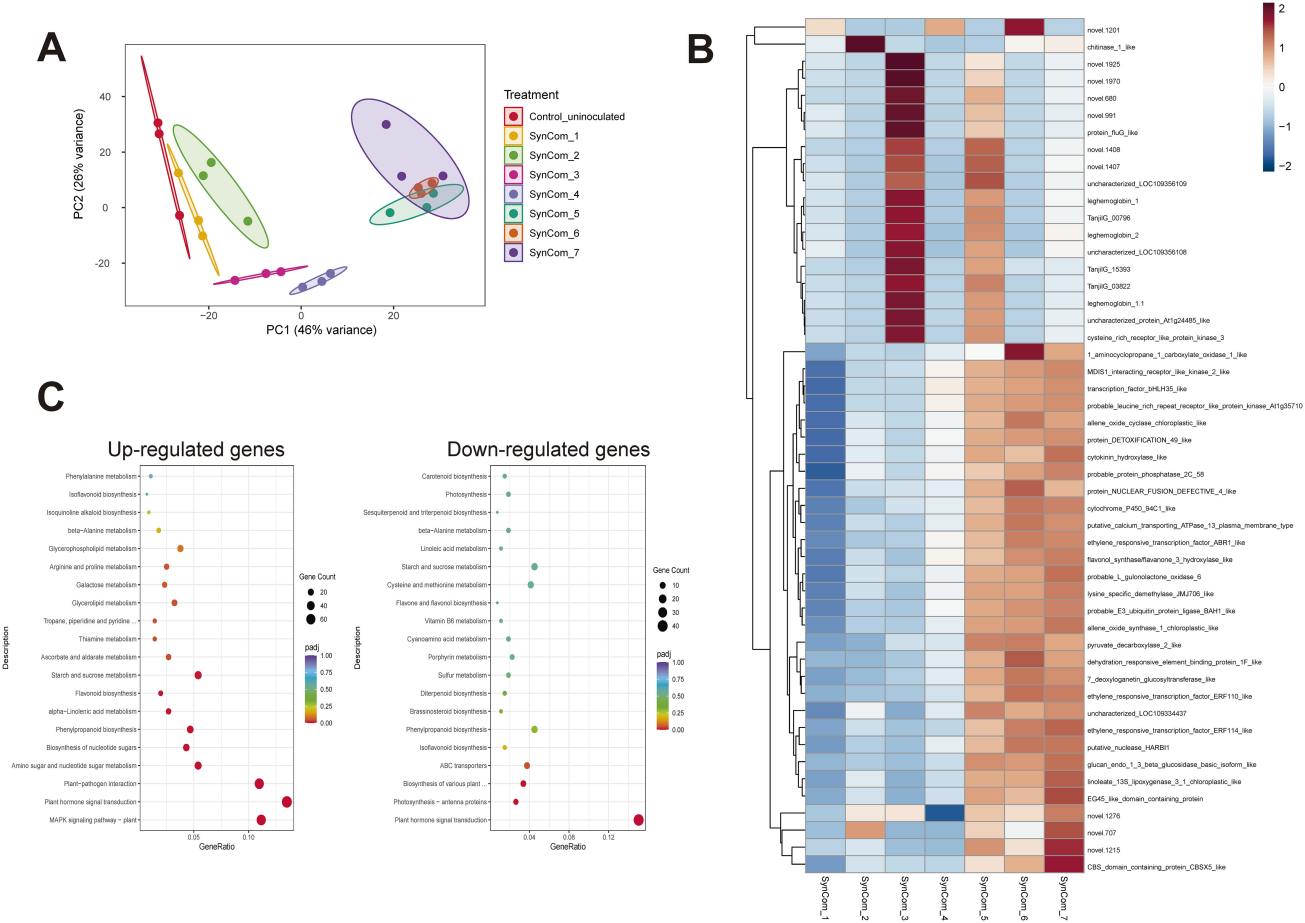
Transcriptomic analysis of lupin plants. **(A)** Principal component analysis (PCA) of transcriptomes from plants inoculated with different SynComs. **(B)** Heatmap showing scaled expression levels of differentially expressed genes in lupins inoculated with SynComs, relative to uninoculated control plants. **(C)** Scatter plot depicting KEGG enrichment analysis of significantly up- and down-regulated genes (P<0.05) in plants treated with SynCom 7 compared to controls.

Principal component analysis (PCA) of the transcriptome data revealed clustering patterns associated with consortia complexity. Control plants clustered closely with those inoculated with a single strain, whereas plants treated with more complex SynComs (e.g., SC-5, SC-6, and SC-7) formed a distinct group (**Figure 6A**). These SynComs induced markedly different gene expression profiles compared to controls, with expression patterns diverging further as bacterial complexity increased (**Figure 6B**).

A notable finding was the overexpression of leghemoglobin genes, suggesting activation of nitrogen fixation-processes activity, particularly under specific microbial interactions. While *Bradyrhizobium* sp. CAR08 alone (SC-1) induced only a slight increase in leghemoglobin expression, its co-inoculation with *Micromonospora* sp. Lupac 08 (SC-3) significantly amplified this response. The inclusion of all selected strains in SC-5 and SC-7 also led to leghemoglobin overexpression, albeit to a lesser extent than in SC-3. However, these treatments were associated with reduced expression of ethylene and putrescine-related genes, alongside increased activation of plant signalling pathways (e.g. MAPK cascade, cytokinin pathways), as well as genes related to stress, senescence, and maturation.

In addition, several defence-related genes were differentially expressed across treatments. Chitinase 1-like, a gene commonly associated with biotic stress responses, was highly expressed in SC-2 but was significantly downregulated depending on SynCom composition. The upregulation of genes such as cysteine-rich receptor-like kinase 3, MDIS1-interacting receptor-like kinase 2-like, and leucine-rich repeat receptor-like kinases in the more complex SynComs (SC-5, -6, and -7) suggests enhanced activation of receptor-mediated signalling pathways. Moreover, increased expression of enzymes involved in hormone metabolism and plant defence, such as allene oxide synthase, lipoxygenase, cytochrome P450, and chitinase 1-like, highlights a coordinated plant response to these microbial communities.

These transcriptional changes were primarily observed when *Bradyrhizobium*, *Micromonospora*, or both were combined with other strains (SC-5, SC-6 and SC-7 respectively). In contrast, in simpler consortia (SC-1 to SC-4) these genes were either downregulated or only weakly upregulated, indicating that the presence of *Micromonospora* and/or *Bradyrhizobium* in more complex communities is critical for full activation of these pathways.

Consistent with these transcriptional patterns, enrichment analysis revealed an overrepresentation of genes involved in hormone signalling, including ethylene-responsive transcription factors (ERF110-like, ERF114-like, ABR1-like), 1-aminocyclopropane-1-carboxylate oxidase, and cytokinin hydroxylase-like, indicating activation of ethylene and cytokinin pathways. The upregulation of transcription factors such as bHLH35-like, Myb4-like, and TIFY 10B-like, together with enzymes such as flavonol synthase, further suggests reprogramming of secondary metabolism, potentially linked to the synthesis of protective compounds.

Additionally, numerous novel and uncharacterized transcripts were identified (e.g. novel.1407, novel.1925, uncharacterized_LOC109356109), indicating that previously unknown genes may contribute to plant responses to microbial interactions and developmental processes. A full list of differentially expressed genes is provided in **Supplementary Table 11**.

Given that SC-7 was the most complex SynCom and led to the highest increase in plant growth, the KEGG pathway analysis provided valuable insights into the underlying molecular mechanisms (**Figure 6C**). Upon addition of SC-7, several key pathways related to signalling, hormone perception, and primary and secondary metabolism were significantly upregulated, including those involved in defence responses and growth regulation. Conversely, certain pathways associated with light harvesting, transport processes, and some aspects of hormone signalling were downregulated, indicating a potential shift in resource allocation away from photosynthetic fine-tuning toward growth and defence.

Similar expression trends were observed in SC-5 and SC-6, whereas SC-1 to SC-4 exhibited markedly different profiles (**Supplementary Figure 20**). Notably, SC-3 displayed the most distinct transcriptional signature among all SynComs. The overexpression of pathways such as plant-pathogen interaction, MAPK signalling pathway (plant), plant hormone signal transduction, carbon fixation, photosynthesis (including antenna proteins), alpha-linolenic acid metabolism, and the pentose phosphate pathway indicates a unique and complex physiological response.

**Figure 7:**
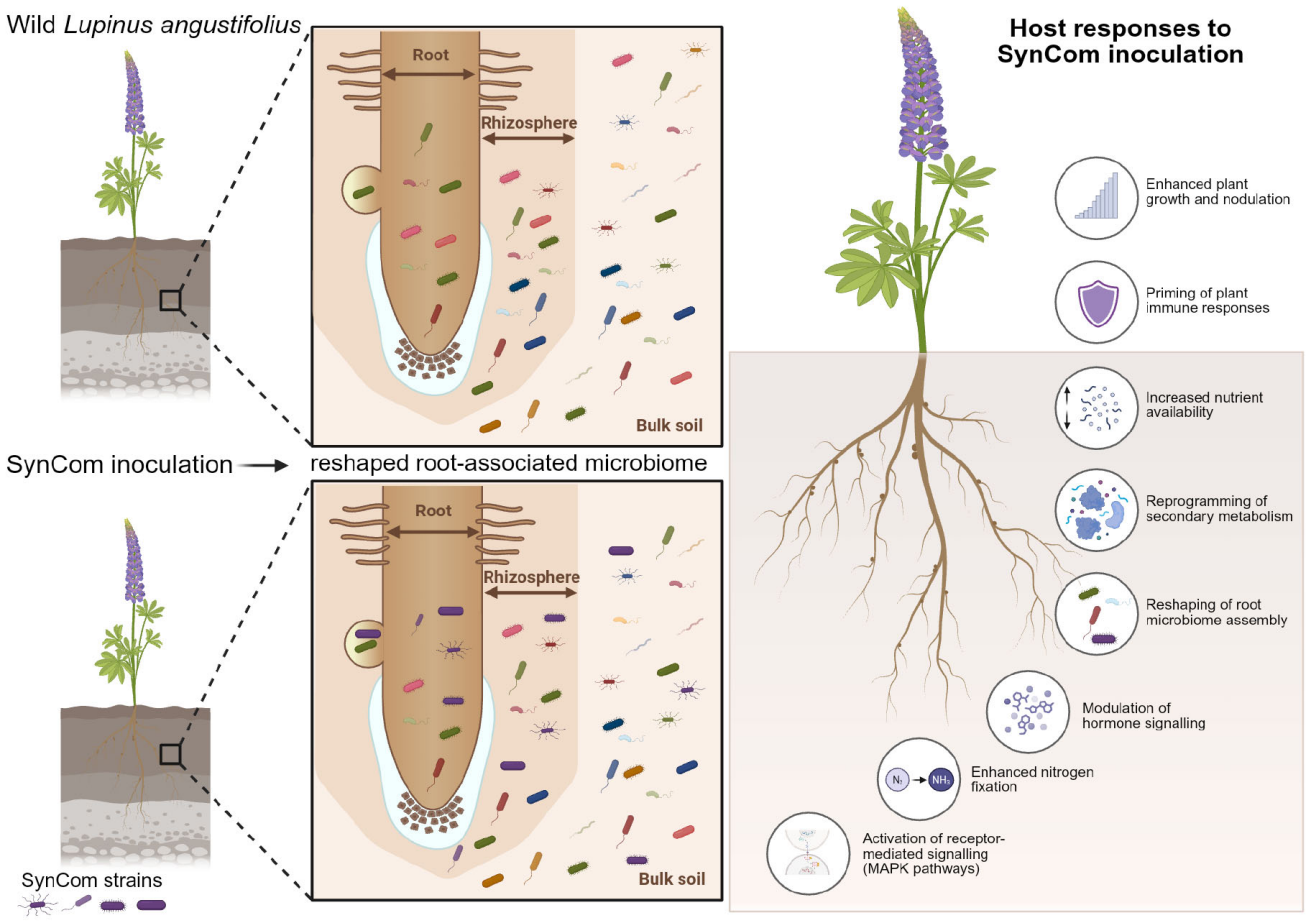
Effects of SynCom on lupin–microbiome interactions. Increasing SynCom complexity is associated with changes in root-associated microbial communities, with moderate effects in the rhizosphere and limited impact on bulk soil microbiota. These microbial shifts co-occur with host transcriptional responses, including the upregulation of genes related to hormone signalling, defence pathways, and metabolic processes. These patterns suggest coordinated plant-microbiome interactions that re associated with improved plant growth performance. Figure created with BioRender. *https://BioRender.com/ztgpty0*.

Overall, these results indicate that increasing SynCom complexity is associated with coordinated changes in plant gene expression, affecting nitrogen fixation, hormone signalling, defence responses, and metabolic processes. These interactions are summarized in a conceptual model illustrating the effects of SynCom composition and environmental conditions in plant–microbiome interactions using *Lupinus* and its associated microbiota as model system (**Figure 7**).

## Discussion

A major limitation in plant microbiome research is the scarcity of indigenous microbial culture collections, which are critical for reconstitution experiments with microbe-free plants to study community assembly and plant interactions. Metagenomic data is valuable for identifying core microbiome members and guiding targeted isolation strategies that can be improved by varying culture media, environmental parameters, and niche-mimicking conditions (Armanhi et al., 2018; Hegazi et al., 2026).

This study provided a comprehensive culture collection associated with *Lupinus angustifolius*, recovering over 700 bacterial strains spanning 87 genera across rhizospheric and endophytic compartments. By integrating metagenomic-informed isolation strategies (Ortúzar et al., 2024) with diverse culture conditions and extended incubation periods, we captured a broad spectrum of microbial diversity, including both dominant and low-abundance taxa (Compant et al., 2019). The marked differences in taxonomic composition across plant compartments, together with the identification of both shared and site-specific genera, highlight the strong spatial structuring of the *L. angustifolius* microbiome and suggest the existence of both conserved core microbiota and environmentally driven community variation.

Across compartments, *Pseudomonas* and *Streptomyces* were consistently detected in the rhizosphere, roots, and leaves, whereas *Agrobacterium*, *Bosea*, *Luteibacter*, and *Rhizobium* were restricted to belowground niches. Notably, *Pseudomonas* was absent from nodules, in contrast to reports in other legumes such as *Cicer*, *Crotolaria*, *Indigofera*, *Lotus*, or *Phaseolus* (Aserse et al., 2013; Crosbie et al., 2022; Yu et al., 2025), suggesting host-specific compartmentalization, niche exclusion, or methodological differences.

Nodules nonetheless exhibited considerable taxonomic richness, with 75 isolates across 22 genera, exceeding diversity reported in other legumes (Martínez-Hidalgo and Hirsch, 2017; Muresu et al., 2019; Brown et al., 2020). The dominance of *Micromonospora* and the restriction of *Bradyrhizobium* to roots and nodules further support the view that nodules host complex and selective microbial assemblages. More broadly, the prevalence of *Pseudomonas*, *Streptomyces*, and *Bacillus*-related taxa across compartments highlights their ecological versatility and frequent association with plants (Bulgarelli et al., 2012; Lundberg et al., 2012; Fitzpatrick et al., 2018; Gates et al., 2023), while the consistent detection of *Bacillus* and *Peribacillus* suggests a potential core microbiota. At the same time, the coexistence of widely distributed and niche-restricted taxa, together with a high proportion of site-specific genera, indicates that both host selection and environmental factors shape community assembly and provides a basis for the rational design of synthetic communities (Dai et al., 2025; Li et al., 2026).

Co-occurrence network analysis provided a working framework to interpret strain selection and SynCom performance, revealing a structured community with distinct topological roles. The identification of hub and connector taxa suggests that community assembly is shaped not only by dominant members but also by highly connected nodes that may contribute to network stability. In this context, several SynCom strains occupied central positions, including *Pseudomonas* (network hub), *Variovorax* (module hub), and connector taxa such as *Rhizobium*, whereas genera such as *Bradyrhizobium*, *Streptomyces*, and *Caulobacter* displayed more peripheral roles, indicative of specialized functions (Agler et al., 2016; Trivedi et al., 2020). The inclusion of strains spanning diverse abundances, phylogenetic groups, and plant compartments supports a design strategy aimed at capturing potential complementary ecological roles. Genomic analyses further reinforced this complementarity, as all strains encoded a broad repertoire of plant growth-promoting traits (PGPTs), particularly those related to vitamin biosynthesis, nutrient acquisition, and plant colonization, while some taxa exhibited more specialized profiles linked to stress mitigation. These findings support the idea that integrating taxa with distinct ecological functions and network positions is critical for reconstructing functional microbial communities and may be associated with enhanced effects observed in more complex SynComs (Delgado-Baquerizo et al., 2025; Wang et al., 2025).

SynCom inoculation in a gnotobiotic system consistently promoted lupin growth, confirming the robustness of microbial effects observed under more complex soil conditions. The superior performance of the most complex consortium (SC-7) suggests that either increased microbial diversity, the presence of particular strains, or both contributed to enhanced plant growth. Because community complexity and strain composition varied simultaneously among SynComs, the individual contributions of these factors cannot be disentangled under the conditions tested. Although all strains successfully colonized plant compartments, their relative abundances varied, indicating that SynCom performance depends not only on strain presence but also on interaction-driven community dynamics (Carlström et al., 2019; Hao et al., 2026).

Microbial community assembly in *Lupinus angustifolius* reflected strong context dependency, driven by both microbe–microbe interactions and the selective environment created at the soil-root interface. Comparison of native and CAS soils revealed marked differences in bacterial composition, with CAS soil dominated by taxa such as *Arthrobacter*, *Massilia*, and *Sphingomonas*, while native soils were enriched in *Gaiella*, *Nitrospira*, *Pseudomonas*, and *Streptomyces*. Despite these differences, microbial communities consistently clustered by plant compartments, with alpha diversity decreasing from soil to rhizosphere to roots, supporting progressive host filtering (Brown et al., 2020; Ortúzar et al., 2024). These differences likely reflect variation in climate, geographic distance, and soil physicochemical properties. Additionally, differences in vegetation cover between sites may further contribute to the observed patterns in soil microbiota (Bahram et al., 2018; Durán et al., 2022). SynCom inoculation had limited impact on bulk soil but significantly influenced rhizosphere and root communities, indicating resistance and functional buffering in established soil microbiota, consistent with ecological theory showing that resident communities constrain invasion through competitive exclusion and niche saturation (Wippel et al., 2021).

Within roots, the SynComs reshaped community composition and drove plant growth promotion. Colonization dynamics were strongly influenced by both microbial interactions and host-mediated effects. For example, *Micromonospora lupini* Lupac08 suppressed *Massilia* in the rhizosphere but promoted its abundance in roots, suggesting context-dependent facilitation or niche opportunities (Trujillo et al., 2015; Naylor et al., 2017; de Souza et al., 2020). Similarly, *Bradyrhizobium* CAR08, acted as a central hub and may contribute to compositional clustering in root microbiomes, potentially through the modulation of root exudation to recruit specific partners (Garrido-Oter et al., 2018; Liu et al., 2019). Other taxa, such as *Pseudomonas*, *Sphingopyxis*, and *Caulobacter*, displayed variable but consistent colonization, while *Streptomyces* and *Micromonospora* were nearly undetectable. Nevertheless, the latter two genera been reported as playing important roles in plant-microbe interactionsdespite the low numbers found in this study (Benito et al., 2017; Martínez-Hidalgo et al., 2014; Santos-Medellín et al., 2021).

Community complexity was also associated with functional outcomes, as more diverse SynComs coincided with greater plant performance and induced broader transcriptional responses than simpler communities. This pattern is consistent with concepts of functional complementarity and redundancy in soil microbial ecosystems (Fitzpatrick et al., 2018; Hassani et al., 2018; Ma et al., 2021) and supports the idea that increased microbial diversity enhances functionality through complementary and overlapping traits (Dai et al., 2025; Ramond et al., 2025). In contrast, simpler communities elicited narrower responses, suggesting that limited diversity constrains functional potential at the soil–root interface. These findings are consistent with broader biodiversity–function relationships, where increased microbial diversity has been associated with enhanced ecosystem multifunctionality and resilience.

Our results show that increasing SynCom complexity was associated with coordinated changes in plant gene expression. PCA (**Figure 6A**) revealed a clear separation between simple and complex consortia, with transcriptional divergence increasing alongside SynCom complexity. This pattern suggests that community complexity is an important factor associated with host transcriptional responses. However as mentioned previously, these effects cannot be attributed exclusively to community complexity, and the contribution of specific strains should also be considered.

An important outcome was the modulation of nitrogen fixation-associated gene expression. Leghemoglobin expression was only weakly induced by *Bradyrhizobium* alone but was strongly enhanced when co-inoculated with *Micromonospora*, suggesting a synergistic interaction. Previous work demonstrated that *Micromonospora* increased nodule number and nitrogen-fixing activity when co-inoculated with *Bradyrhizobium* (Benito et al., 2017). Here, we show that this synergistic effect is maintained even in the presence of additional bacterial species. Complex SynComs further triggered extensive reprogramming of signalling and defence pathways. The upregulation of genes involved in MAPK cascades, cytokinin signalling, and receptor-like kinases, together with changes in ethylene-related genes, suggests a shift in hormonal balance and enhanced microbial perception. These responses were largely restricted to consortia containing *Micromonospora* and/or *Bradyrhizobium*, highlighting the importance of community context.

Functional analyses revealed a broad metabolic reprogramming characterized by activation of hormone signalling genes, defence responses, and secondary metabolism, alongside downregulation of photosynthesis-related pathways. This pattern indicates a reallocation of plant resources toward growth and defence under complex microbial conditions. In line with this, SC-3 exhibited a distinct transcriptional profile, supporting the notion that specific microbial combinations can generate unique, non-additive effects. Moreover, SynCom inoculation primarily influenced root-associated microbial communities, with limited impact on bulk soil, reinforcing the role of the root as the central interface for plant–microbiome interactions.

This work demonstrates associations between SynCom complexity, plant performance, microbiota assembly, and functional traits in soil–plant–microbe systems. They corroborate previous observations of incomplete colonization by low-abundance taxa and highlight the potential contribution of both dominant and rare biosphere members in shaping community dynamics and plant stress responses. The pronounced effects within the root environment further emphasize the importance of synchronizing microbial applications with key plant developmental stages. From an applied perspective, translating these insights into agricultural practice will require the rigorous design, selection, and validation of microbial consortia under field-realistic conditions. In this context, the newly established strain collection provides a valuable resource for constructing more complex synthetic communities and advancing our understanding of plant microbiome assembly.

## Data Availability

The sequencing data from this study have been deposited in the National Center for Biotechnology information Bioproject database (https://www.ncbi.nlm.nih.gov/bioproject/) under accession numbers PRJNA1178901, PRJNA1335402, and PX430780 – PX431494.
