# Supplementary material for: From natural assemblages to synthetic communities in the Lupinus microbiome
Source: Front Plant Sci. 2026 Jul 31;17:1891479. doi: 10.3389/fpls.2026.1891479 (PMC13473416; doi:10.3389/fpls.2026.1891479)
Supplement: Supplementary file 1 [file DataSheet1.docx]

**SUPPLEMENTARY MATERIAL**

**From natural assemblages to synthetic communities in the *Lupinus* microbiome**

Maite Ortúzar^1^*, Víctor Formariz^1^, Jhon Alexander Suescún-Sepúlveda^1^, Marta González-Hernández^1^, Raúl Riesco^1^, Ruben Garrido-Oter^2,3,4^, Martha E. Trujillo^1^*

^1^Departamento de Microbiología y Genética, University of Salamanca, Salamanca, Spain

^2^Department of Plant-Microbe Interactions, Max Planck Institute for Plant Breeding Research, Cologne, Germany

^3^Cluster of Excellence on Plant Sciences, Düsseldorf, Germany

^4^Earlham Institute, Norwich, UK

**Corresponding authors:**

Martha E. Trujillo, email: [mett@usal.es](mailto:mett@usal.es); Maite Ortúzar, email: [maiteortuzar@usal.es](mailto:maiteortuzar@usal.es)

**SUPPLEMENTARY FIGURES**

**Figure S1. Root-associated core microbiome. (A)** Venn diagram showing the genera shared across locations. Taxa were defined as core members when detected in at least 90% of samples within each location and exhibiting a relative abundance greater than 0.1%. **(B)** Bar plot showing the relative abundance of the core genera.

**Figure S2. Plant inoculation experiments.**  **(A)** Experimental design of SynCom inoculation under gnotobiotic conditions. **(B)** Experimental design assessing the effect of SynComs on the bacterial community in a non-sterile soil.

**Figure S3. Distribution of the bacterial isolate collection according to culture medium, sampling location and plant compartment. (A)** Proportion of isolates recovered across different culture media (ISP2, NA, PYE, R2A, SCA, and YMA). **(B)** Distribution of isolates by sampling location (Cabrerizos and Salamanca). **(C)** Distribution of isolates across plant compartments (rhizosphere, roots, nodules, and leaves).

**Figure S4. Venn diagram showing the distribution of bacterial genera across sampling sites and isolation sources. (A)** Percentage of shared and unique genera between the two sampling sites (Cabrerizos vs. Salamanca). **(B)** Distribution of bacterial genera by isolation source (rhizosphere, roots, nodules, and leaves).

**Figure S5. Maximum-likelihood phylogenetic tree of the phylum *Actinomycetota* based on 16S rRNA gene sequences.** The tree depicts family level relationships among all *Actinomycetota* strains isolated in this study, annotated by plant compartments (leaves, nodules, rhizosphere and roots), and sampling location. Evolutionary distances were calculated using the Kimura two- parameter model. Bootstrap support values ≥90 % (1,000 replicates) are indicated at the nodes. Scale bar, 0.1 substitutions per nucleotide position.

**Figure S6.** **Maximum-likelihood phylogenetic tree of the phylum *Bacillota* based on 16S rRNA gene sequences**. The tree depicts family level relationships among all *Bacillota* strains isolated in this study, annotated by plant compartments (leaves, nodules, rhizosphere and roots), and sampling location. Evolutionary distances were calculated using the Kimura two-parameter model. Bootstrap support values ≥90% (1,000 replicates) are indicated at the nodes. Scale bar, 0.1 substitutions per nucleotide position.

**Figure S7.** **Maximum-likelihood phylogenetic tree of the phylum *Bacteroidota* based on 16S rRNA gene sequences.** The tree depicts family-level relationships among all *Bacteroidota* strains isolated in this study, annotated by plant compartment (leaves, nodules, rhizosphere, and roots) and sampling location. Evolutionary distances were calculated using the Kimura two-parameter model. Bootstrap support values ≥90% (1,000 replicates) are indicated at the nodes. Scale bar, 0.1 substitutions per nucleotide position.

**Figure S8.** **Maximum-likelihood phylogenetic tree of the phylum *Pseudomonadota* based on 16S rRNA gene sequences.** The tree depicts family family level of all *Pseudomonadota* strains isolated in this study, annotated by plant compartments (leaves, nodules, rhizosphere and roots), and sampling location. Evoltuionary distances were calculated using the Kimura two- parameter model. Bootstrap support values ≥90 % (1,000 samplings) are indicated at nodes. Scale bar, 0.1 substitutions per nucleotide position.

**Figure S9. Recovery rates of isolates and their relationship with metagenomic abundance. (A)** Scatter plots showing the relationship between relative abundance in 16S rRNA-derived metagenomic profiles and the number of cultured isolates across samples. **(B)** Heatmap showing recovery rates (Observed/Expected) across genera and plant compartments.

**Figure S10. Distribution and overlap of bacterial genera across plant compartments.** Bar plot showing shared bacterial genera between the rhizosphere and plant tissues from which the strains were isolated.

**Figure S11. Comparison of bacterial community composition inferred from 16S rRNA gene amplicon sequencing and the corresponding cultured isolates. (A)** Community composition assessed by 16S rRNA gene amplicon sequencing. Sequences with relative abundances >1% are clustered at the class, family and genus levels. Bar heights represent taxon relative abundances *in situ*. **(B)** Number of isolated bacterial strains corresponding to each taxon. Bar heights indicate strain counts. Shared genera in both amplicon data and culture collection are connected via lines.

**Figure S12. Evaluation of the pathogenic potential and antagonistic activity of the isolated strains. (A)** Classification of the 722 strains into non-pathogens, plant growth-promoting (PGP), and potential pathogens. **(B)** Distribution of potential pathogen strains across different isolation compartments (leaves, nodules, rhizosphere and roots). **(C)** Genus-level distribution of strains identified as potential pathogens. **(D)** Representative images from pairwise antagonism assays performed on agar plates.

**Figure S13.** **Plant growth parameters and performance in a gnotobiotic system**. Images of plants inoculated with different SynComs compared with uninoculated controls. Quantitative plant growth parameters (shoot length (cm), shoot fresh weight (g), root fresh weight (g), and number of nodules) were standardized using Z-scores to enable comparison across traits and are shown as box plots (Tukey P<0.001). Data are presented for two independent experiments (**A** and **B,** respectively). Raw measurements of all plant growth parameters are provided in Table S6.

**Figure S14. Abundance of strains detected in the host plant.** **(A)** Relative abundance of individual strains within different SynComs across two compartments: rhizosphere and roots. Detailed abundances are shown for the **(B)** rhizosphere, and **(C)** roots.

**Figure S15. Bacterial community composition and structure**. **(A)** Bar plot showing the relative abundance of bacterial taxa based on 16S rRNA gene amplicon sequencing of *Lupinus* plants grown under greenhouse conditions in three soils: Salamanca, Cabrerizos, and CAS (Cologne Agricultural Soil). **(B)** Principal coordinate analysis (PCoA) of the same samples.

**Figure S16.** **Alpha diversity of soil and rhizosphere bacterial communities in a natural community soil**. Box plots of the Shannon diversity index (alpha diversity) showing the effects of different SynComs on bacterial communities in **(A)** soil and **(B)** rhizosphere samples. Different letters above the box plots indicate statistically significant differences among groups (P<0.001).

**Figure S17. Alpha diversity of soil and rhizosphere bacterial communities in natural community soil following *in silico* removal of SynCom-associated ASVs.** Box plots of the Shannon diversity index (alpha diversity) showing the effects of different SynComs on bacterial communities in **(A)** soil and **(B)** rhizosphere samples. Different letters above the box plots indicate statistically significant differences among groups (*P* < 0.001).

**Figure S18.** **Effect of SynCom inoculation on the natural bacterial community associated with the host plant following *in silico* removal of SynCom ASVs**. **(A)** Box plots showing the Shannon diversity index (alpha diversity) of bacterial communities in soil, rhizosphere and roots following inoculation with different SynComs. **(B)** Detailed comparison of the Shannon index in root bacterial communities following inoculation with different SynComs. Different letters above the box plots indicate statistically significant differences among groups (P<0.001). **(C)** Principal coordinates analysis (PCoA) based on Bray-Curtis dissimilarities (beta diversity) showing differences in bacterial community composition across soil, rhizosphere, and root samples inoculated with different SynComs. Group differences were assessed using PERMANOVA (P<0.001). **(D)** Bar plots showing the relative abundance of the most prevalent bacterial genera in the soil, rhizosphere, and roots following inoculation with different SynComs.

**Figure S19. Plant growth parameters in non-sterilized soil.** Representative images of plants inoculated with different SynComs compared with uninoculated controls. Quantitative plant growth parameters (shoot length, shoot fresh weight, root fresh weight, and number of nodules) were standardized as Z-scores to enable comparison across traits and are represented as box plots. Different letters indicate statistically significant difference among groups (Tukey P<0.001). Data is presented for two independent experiments (**A** and **B**, respectively). Raw measurements of all plant growth parameters are provided in Table S6.

**Figure S20. KEGG pathway enrichment analysis of transcriptomic data.**  **(A)** Scatter plot of enriched KEGG pathways for significantly up-regulated genes (P < 0.05), and **(B)** for significantly downregulated genes, in plants treated with different SynComs relative to control plants.
